# Constraint and Contingency in Multifunctional Gene Regulatory Circuits

**DOI:** 10.1371/journal.pcbi.1003071

**Published:** 2013-06-06

**Authors:** Joshua L. Payne, Andreas Wagner

**Affiliations:** 1 University of Zurich, Institute of Evolutionary Biology and Environmental Studies, Zurich, Switzerland; 2 Swiss Institute of Bioinformatics, Lausanne, Switzerland; 3 Bioinformatics Institute, Agency for Science, Technology, and Research (A*STAR), Queenstown, Singapore; 4 The Santa Fe Institute, Santa Fe, New Mexico, United States of America; Pennsylvania State University, United States of America

## Abstract

Gene regulatory circuits drive the development, physiology, and behavior of organisms from bacteria to humans. The phenotypes or functions of such circuits are embodied in the gene expression patterns they form. Regulatory circuits are typically multifunctional, forming distinct gene expression patterns in different embryonic stages, tissues, or physiological states. Any one circuit with a single function can be realized by many different regulatory genotypes. Multifunctionality presumably constrains this number, but we do not know to what extent. We here exhaustively characterize a genotype space harboring millions of model regulatory circuits and all their possible functions. As a circuit's number of functions increases, the number of genotypes with a given number of functions decreases exponentially but can remain very large for a modest number of functions. However, the sets of circuits that can form any one set of functions becomes increasingly fragmented. As a result, historical contingency becomes widespread in circuits with many functions. Whether a circuit can acquire an additional function in the course of its evolution becomes increasingly dependent on the function it already has. Circuits with many functions also become increasingly brittle and sensitive to mutation. These observations are generic properties of a broad class of circuits and independent of any one circuit genotype or phenotype.

## Author Summary

Many essential biological processes, ranging from embryonic patterning to circadian rhythms, are driven by gene regulatory circuits, which comprise small sets of genes that turn each other on or off to form a distinct pattern of gene expression. Gene regulatory circuits often have multiple functions. This means that they can form different gene expression patterns at different times or in different tissues. We know little about multifunctional gene regulatory circuits. For example, we do not know how multifunctionality constrains the evolution of such circuits, how many circuits exist that have a given number of functions, and whether tradeoffs exist between multifunctionality and the robustness of a circuit to mutation. Because it is not currently possible to answer these questions experimentally, we use a computational model to exhaustively enumerate millions of regulatory circuits and all their possible functions, thereby providing the first comprehensive study of multifunctionality in model regulatory circuits. Our results highlight limits of circuit designability that are relevant to both systems biologists and synthetic biologists.

## Introduction

Gene regulatory circuits are at the heart of many fundamental biological processes, ranging from developmental patterning in multicellular organisms [1] to chemotaxis in bacteria [2]. Regulatory circuits are usually multifunctional. This means that they can form different metastable gene expression states under different physiological conditions, in different tissues, or in different stages of embryonic development. The segment polarity network of *Drosophila melanogaster* offers an example, where the same regulatory circuit affects several developmental processes, including embryonic segmentation and the development of the fly's wing [3]. Similarly, in the vertebrate neural tube, a single circuit is responsible for interpreting a morphogen gradient to produce three spatially distinct ventral progenitor domains [4]. Other notable examples include the bistable competence control circuit of *Bacillus subtilis* [5] and the lysis-lysogeny switch of bacteriophage lambda [6]. Multifunctional regulatory circuits are also relevant to synthetic biology, where artificial oscillators [7], toggle switches [8], and logic gates [9] are engineered to control biological processes.

The functions of gene regulatory circuits are embodied in their gene expression patterns. An important property of natural circuits, and a design goal of synthetic circuits, is that these patterns should be robust to perturbations. Such perturbations include *nongenetic* perturbations, such as stochastic fluctuations in protein concentrations and environmental change. Much attention has focused on understanding [1], [2], [4], [10], [11] and engineering [12]–[14] circuits that are robust to nongenetic perturbations. Equally important is the robustness of circuit functions to *genetic* perturbations, such as those caused by point mutation or recombination. Multiple studies have asked what renders biological circuitry robust to such genetic changes [15]–[20]. With few exceptions [21], [22], these studies have focused on circuits with one function, embodied in their gene expression pattern. Such monofunctional circuits tend to have several properties. First, many circuits exist that have the same gene expression pattern [17]–[19], [23]–[28]. Second, these circuits can vary greatly in their robustness [16], [18], [29]. And third, they can often be reached from one another via a series of function-preserving mutational events [18], [19], [30]. Taken together, these observations suggest that the robustness of the many circuits with a given regulatory function can be tuned via incremental mutational change.

Most circuits have multiple functions, but how these observations translate to such multifunctional circuits is largely unknown. In a given space of possible circuits, how many circuits exist that have a given number of *k* specific functions (expression patterns)? What is the relationship between this number of functions and the robustness of each function? Do circuits with any combination of functions exist, or are some combinations “prohibited?” Pertinent earlier work showed that there are indeed fewer multifunctional circuits than monofunctional circuits [21], but this investigation had two main limitations. First, it considered circuits so large that the space of circuits and their functions could not be exhaustively explored, and restricted itself to mostly bifunctional circuits. Second, it included only *topological* circuit variants (*i.e.*, who interacts with whom), and ignored variations in the signal-integration logic of *cis*-regulatory regions. These regions encode regulatory programs, which specify the input-output mapping of regulatory signals (input) to gene expression pattern (output) [31]–[33]. Variations in *cis*-regulatory regions [34], such as mutations that change the spacing between transcription factor binding sites [35], are known to impact circuit function [36], [37], and their inclusion in a computational model of regulatory circuits is thus important.

Here, we overcome these limitations by focusing on regulatory circuits that are sufficiently small that an entire space of circuits can be exhaustively explored. Specifically, we focus on circuits that comprise only three genes and all possible regulatory interactions between them. Small circuits like this play an important role in some biological processes. Examples include the *kaiABC* gene cluster in Cyanobacteria, which is responsible for circadian oscillations [38], the gap gene system in *Dropsophila*, which is responsible for the interpretation of morphogen gradients during embryogenesis [19], and the *krox-otx-gatae* feedback loop in starfish, which is necessary for endoderm specification [39]. Additionally, theoretical studies of small regulatory circuits have provided several general insights into the features of circuit design and function. Examples include biochemical adaptation in feedback loops [40] and response delays in feed-forward loops [41], among others [16], [19], [23], [42]–[45]. Lastly, there is a substantial body of evidence suggesting that small regulatory circuits form the building blocks of larger regulatory networks [34], [46]–[48], further warranting their study.

For two reasons, we chose Boolean logic circuits [49] as our modeling framework. First, they allow us not only to vary circuit topology [45], but also a circuit's all-important signal-integration logic [44]. Second, Boolean circuits have been successful in explaining properties of biological circuits. For example, they have been used to explain the dynamics of gene expression in the segment polarity genes of *Drosophila melanogaster* [50], the development of primordial floral organ cells of *Arabidopsis thaliana* [51], gene expression cascades after gene knockout in *Saccharomyces cerevisiae* [52], and the temporal and spatial expression dynamics of the genes responsible for endomesoderm specification in the sea urchin embryo [53]. We consider a specific gene expression pattern as the function of a circuit like this, because it is this pattern that ultimately drives embryonic pattern formation and physiological processes. Multifunctional circuits are circuits with multiple gene expression patterns, and here we study the constraints that multifunctionality imposes on the robustness and other properties of regulatory circuits. The questions we ask include the following: (i) How many circuits have a given number *k* of functions? (ii) What is the relationship between multifunctionality and robustness to genetic perturbation? (iii) Are some multifunctional circuits more robust than others? (iv) Is it possible to change one multifunctional circuit into another through a series of small genetic changes that do not jeopardize circuit function?

## Results

### The model

**Figure 1:**
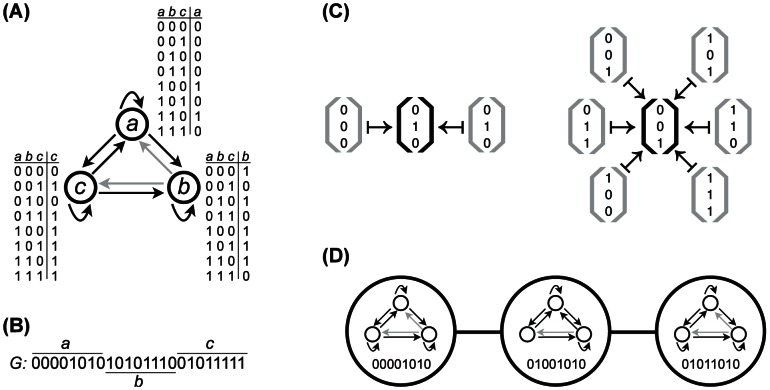
Schematic illustration of the Boolean model of gene regulatory circuits. (A) A Boolean circuit with 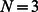 genes (*a,b,c*), which are represented as open circles. Two genes are connected by a directed edge 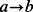 if the expression of gene *b* is regulated by the product of gene *a*. Gene expression is binary, such that genes are either expressed (1) or not (0). The signal-integration logic of each gene is shown as a lookup table that explicitly maps all 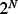 possible input expression states to an output expression state, implicitly determining the circuit's topology. In the hypothetical circuit shown, the expression state of gene *a* is independent of the expression state of gene *b*, so 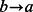 is a non-existing regulatory interaction (gray arrow), whereas 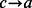 and 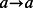 are both existing regulatory interactions (black arrows). (B) The wiring diagram and signal-integration logic of the entire circuit can be represented by a single vector *G* that is constructed by concatenating the rightmost columns of the lookup tables of the individual genes in panel (A). The vector *G* corresponds to the circuit's genotype. (C) The circuit in (A) maps all of the 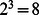 possible initial states 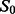 (gray brackets) onto two distinct stable equilibrium expression states 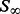 (black brackets). This circuit therefore can have up to 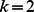 functions, and can express such a “bifunction” in 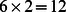 different ways, since 6 initial states map to one equilibrium expression state and the other 2 initial states map to another equilibrium expression state. (D) In a genotype network, vertices represent circuits and two vertices share an edge if the genotypes *G* differ by a single element, yet have the same functions. Here, the genotype network corresponds to circuits with the bifunction 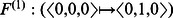, 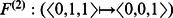. For visual clarity, each circle only shows the first 8 binary digits of *G*, which represent the signal-integration logic of gene *a*. Note how changes in *G* may implicitly translate to changes in circuit topology.

We consider circuits of 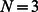 genes (Fig. 1A). We choose a compact representation of a circuit's genotype *G* that allows us to represent both a circuit's signal-integration logic and its architecture by a single binary vector of length 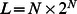 (Fig. 1B). Changes to this vector can be caused by mutations in the *cis*-regulatory regions of DNA. Such mutations may alter the binding affinity of a transcription factor to its binding site, thereby creating or removing a regulatory interaction [34]. Alternatively, they may affect the distance of a transcription factor binding site from the transcription start site, changing its rotational position on the DNA helix. In turn, this may alter the regulatory effect of the transcription factor [54], and change the downstream gene's signal-integration logic. Lastly, such mutations may change the distance between adjacent transcription factor binding sites, enabling or disabling a functional interaction between proximally bound transcription factors [35]. We note that mutations in *G* could also be conceptualized as changes in the DNA binding domain of a transcription factor. However, evolutionary evidence from microbes suggest that alterations in the structure and logic of regulatory circuits occurs preferentially via changes in *cis*-regulatory regions, rather than via changes in the transcription factors that bind these regions [55].

The dynamics of the expression states of a circuit's *N* genes begin with a prespecified initial state 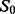, which represents regulatory influences outside or upstream of the circuit, such as transcription factors that are not part of the circuit but can influence its expression state. The initial state reflects the fact that small circuits are typically embedded in larger regulatory networks [34], [46]–[48], which provide the circuit with different regulatory inputs under different environmental or tissue-specific conditions. Through the regulatory interactions specified in the circuit's genotype, the circuit's gene expression state changes from this initial state, until it may reach a stable (*i.e.*, fixed-point) equilibrium state 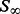. We consider a circuit's function to be a mapping from an initial expression state to an equilibrium expression state 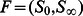 (Fig. 1C). In the main text, we consider only circuit functions that involve fixed point equilibria, but we consider periodic equilibrium states in the Supporting Online Material. A circuit could in principle have as many as 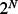 functions 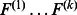, as long as the initial expression states are all different from one another, and the equilibrium expression states are all different from one another (Material and Methods). The circuits we study may map multiple initial states to the same equilibrium state, but our definition of function ignores all but one of these initial states. While a definition of function that includes many-to-one mappings between initial and equilibrium states can be biologically sensible, our intent is to investigate specific pairs of inputs (*i.e.*, 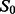) and outputs (*i.e.*, 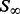), as is typical for circuits in development and physiology [56]–[58]. We emphasize that a circuit can express its *k* functions individually, or in various combinations, such that the same circuit could be said to have between one and *k* functions. For brevity, we refer to a specific set of *k* functions as a multifunction or a *k*-function and to circuits that have at least one function as viable.

The space of circuits we explore here contains 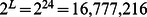 possible genotypes. We exhaustively determine the equilibrium expression states of each genotype for all 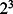 initial states, thereby providing a complete genotype-to-phenotype(function) map. We use this map to partition the space of genotypes into genotype networks [17]–[19], [21]. A genotype network consists of a single connected set of genotypes (circuits) that have identical functions 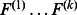, and where two circuits are connected neighbors if their corresponding genotypes differ by a single element (Fig. 1D). Note that such single mutations may correspond to larger mutational changes in the *cis*-regulatory regions of DNA. For example, mutations that change the distance between binding sites, or between a binding site and a transcription start site, may involve the addition or deletion of large segments of DNA [26], [59]–[62].

### Multifunctionality constrains the number of viable circuits

**Figure 2:**
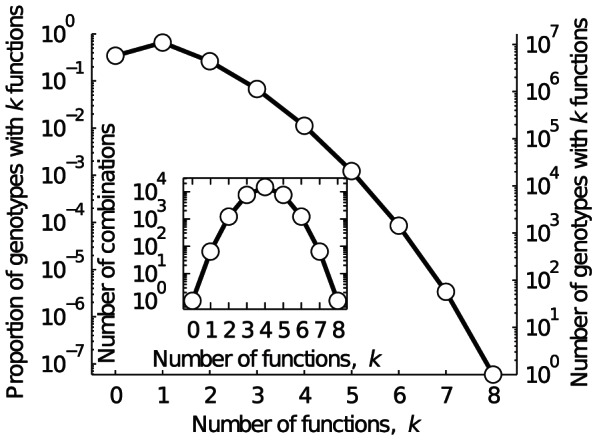
Multifunctional regulatory circuits. Each data point depicts the proportion and number of genotypes with *k* functions. The data include all *k*-functions. The line is provided as a visual guide. Note that there are more circuits with 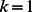 function than with 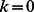 functions, implying that a randomly selected circuit is more likely to be viable than not. Also note that any circuit with *k* functions will be included in the count of the number of circuits with between 1 and 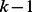 functions. The inset shows the number of observed combinations of functions (open circles) and the total number of possible combinations (solid line) of *k* functions. Note the logarithmic scale of all y-axes.

We first asked how the number of genotypes that have *k* functions depends on *k*. Fig. 2 shows that this number decreases exponentially, implying that multifunctionality constrains the number of viable genotypes severely. For instance, increasing *k* from 1 to 2 decreases the number of viable genotypes by 34%; further increasing *k* from 2 to 3 leads to an additional 39% decrease. However, there is always at least one genotype with a given number *k* of functions, for any 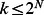. In other words, even in these small circuits, multiple genotypes exist that have many functions.

Thus far, we have determined the number of genotypes with a given number *k* of functions, but we did not distinguish between the actual functions that these genotypes can have. For example, there are 64 variants of 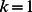 function, since there are 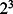 potential initial states and 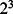 potential equilibrium states (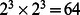). Analogously, simple combinatorics (Text S1) shows that there are 1204 variants of 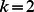 functions, and the number of variants increases dramatically with greater *k*, up to a maximum of 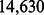 variants of 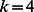 functions. This is possible because individual functions can occur in different possible combinations in multifunctional circuits (Material and Methods). The solid line in the inset of Fig. 2 indicates how this number of possible different functions scales with *k*. We next asked whether there exist circuits (genotypes) for each of these possible combinations of functions, or whether some multifunctions are prohibited. The open circles in the inset of Fig. 2 show the answer: These circles lie exactly on the solid line that indicates the number of possible combinations of functions for each value of *k* (Text S1). This means that no multifunction is prohibited. In other words, even though multifunctionality constrains the number of viable genotypes, there is always at least one genotype with *k* functions, and in any possible combination.

### A trade-off between multifunctionality and robustness

As gene regulatory circuits are often involved in crucial biological processes, their functions should be robust to perturbation. We therefore asked whether the constraints imposed by multifunctionality also impact the robustness of circuits and their functions. In studying robustness, we differentiate between the robustness of a genotype (circuit) and the robustness of a *k*-function. We assess the robustness of a genotype as the proportion of all possible single-mutants that have the same *k*-function, and the robustness of a *k*-function as the average robustness of all genotypes with that *k*-function [17], [18], [51], [63] (Material and Methods). We refer to the collection of genotypes with a given *k*-function as a genotype set, which may comprise one or more genotype networks. We emphasize that a genotype may be part of several different genotype sets, because genotypes typically have more than one *k*-function.

**Figure 3:**
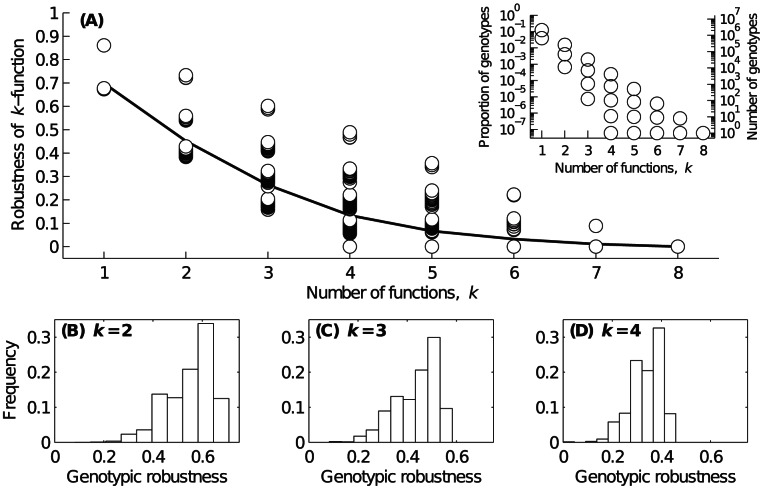
Robustness and multifunctionality. (A) The robustness of a *k*-function is shown in relation to the number of functions *k*. Each data point corresponds to the genotype set of a specific combination of *k* functions. The data include all *k*-functions. The solid line depicts the average robustness of a *k*-function. The inset shows the proportion and number of genotypes in the genotype set of a *k*-function, as a function of *k*. Note the logarithmic scale of the y-axes. (B–D) Distributions of genotypic robustness for (B) 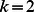, (C) 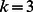, and (D) 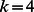. For each *k*, we show data for a single genotype network.

Fig. 3A shows that the robustness of a *k*-function decreases approximately linearly as *k* increases, indicating a trade-off between multifunctionality and robustness. However, some degree of robustness is maintained so long as 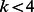. For larger *k*, some functions exist that have zero robustness (Text S1), that is, none of the circuits with these functions can tolerate a change in their regulatory genotype. The inset of Fig. 3A reveals a similar inverse relationship between the size of a genotype set and the number of functions *k*, implying that multifunctions become increasingly less “designable” [64] — fewer circuits have them — as *k* increases (Text S1). For example, for as few as 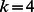 functions, the genotype set may comprise a single genotype, reducing the corresponding robustness of the *k*-function to zero. For each value of *k*, the maximum proportion of genotypes with a given *k*-function is equal to the square of the maximum proportion of genotypes with a 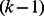 function, explaining the triangular shape of the data in the inset. This triangular shape indicates that the genotype set of a given *k*-function is always smaller than the union of the *k* constituent genotypes sets. Additionally, we find that the robustness of a *k*-function and the size of its genotype set are strongly correlated (Fig. S1), indicating that the genotypes of larger genotype sets are, on average, more robust than those of smaller genotype sets. This result is not trivial because the structure of a genotype set may change with its size. For example, large genotype sets may comprise many isolated genotypes, or their genotype networks might be structured as long linear chains. In either case, the robustness of a *k*-function would decrease as the size of its genotype set increased.

We have so far focused on the properties of the genotype sets of *k*-functions, but have not considered the properties of the genotype networks that make up these sets. Therefore, we next asked how genotypic robustness varies across the genotype networks of *k*-functions. In Figs. 3B–D, we show the distributions of genotypic robustness for representative genotype networks with 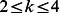 functions. These distributions highlight the inherent variability in genotypic robustness that is present in the genotype networks of multifunctions, indicating that genotypic robustness is an evolvable property of multifunctional circuits. Indeed, in Fig. S2, we show the results of random walks on these genotype networks, which confirm that it is almost always possible to increase genotypic robustness through a series of mutational steps that preserve the *k*-function. In Fig. S3, we show in which dynamic regimes (Material and Methods) the circuits in these same genotype networks lie.

### Multifunctionality leads to genotype set fragmentation

We have shown that the genotype set of any *k*-function is non-empty (Fig. 2), meaning that there are no “prohibited” *k*-functions. We now ask how the genotypes with a given *k*-function are organized in genotype space. More specifically, is it possible to connect any two circuits with the same *k*-function through a sequence of small genotypic changes where each change in the sequence preserves this *k*-function? In other words, are all genotypes with a given *k*-function part of the same genotype network, or do such genotypes occur on multiple disconnected genotype networks?

**Figure 4:**
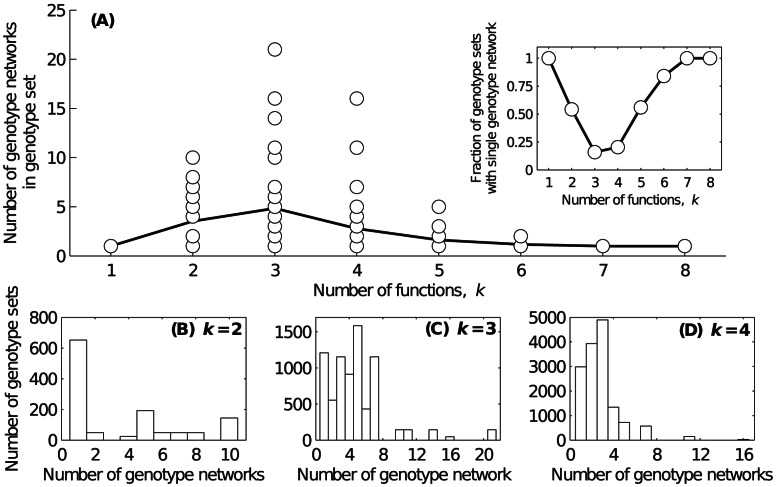
Genotype set fragmentation. (A) Each data point shows the number of genotype networks in the genotype set of a specific *k*-function. The data include all *k*-functions. The solid line depicts the average number of genotype networks per *k*-function. The inset shows the proportion of genotype sets that comprise a single genotype network, as a function of *k*. (B–D) The distributions of the number of genotype networks per genotype set for (B) 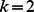, (C) 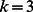, and (D) 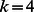.

Fig. 4 shows the relationship between the number of genotype networks in a genotype set and the number of circuit functions *k*. For monofunctional circuits (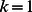), the genotype set always consists of a single, connected genotype network. This implies that any genotype in the genotype set can be reached from any other via a series of function-preserving mutational events. In contrast, for circuits with 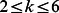 functions, the genotype set often fragments into several isolated genotype networks, indicating that some regions of the genotype set cannot be reached from some others without jeopardizing circuit function. The most extreme fragmentation occurs for 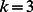 functions, where some genotype sets break up into more than 20 isolated genotype networks. Fig. S4 provides a schematic illustration of how fragmentation can occur in a *k*-function's genotype set, despite the fact that the genotype sets of the *k* constituent monofunctions consist of genotype networks that are themselves connected. Fig. S5 provides a concrete example of fragmentation, depicting one genotype from each of the several genotype networks of a bifunction's genotype set.

The proportion of *k*-functions with genotype sets that comprise a single genotype network is shown in the inset of Fig. 4. This proportion decreases dramatically as the number of functions increases from 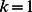 to 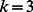, such that only 16% of genotype sets comprise a single genotype network when 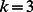. Figs. 4B–D show that the distributions of the number of genotype networks per genotype set are typically left-skewed. This implies that when fragmentation occurs, the genotype set usually fragments into only a few genotype networks. However, the distribution of genotype network sizes across all genotype sets is heavy-tailed and often spans several orders of magnitude (Fig. S6). This means that the number of genotypes per genotype network is highly variable.

We next ask whether the number of genotypes in the genotype set of a *k*-function can be predicted from the number of genotypes in the genotype sets of the *k* constituent monofunctions. To address this question, we define the fractional size of a genotype set as the number of genotypes in the set, divided by the number of genotypes in genotype space. We first observe that the maximum fractional size of a genotype set of a *k*-function is equal to 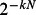 (Fig. S6), which is the maximum fractional size of a genotype set for monofunctional circuits [44] raised to the *k*th power. In general, we find that the fractional size of a genotype set of a *k*-function can be approximated with reasonable accuracy by the product of the fractional sizes of the genotype sets of the *k* constituent monofunctions, but that the accuracy of this approximation decreases as *k* increases (Fig. S7). While these fractional genotype set sizes may be quite small, we note that their absolute sizes are still fairly large, even in the tiny circuits considered here. For example, for 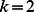 functions the maximum genotype set size is 262,144. For 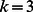 functions, the maximum is 32,768.

### Genotype set fragmentation may lead to historical contingency

In evolution, a circuit may acquire a new regulatory function while preserving its pre-existing functions. An example is the highly-conserved *hedgehog* regulatory circuit, which patterns the insect wing blade. In butterflies, this regulatory circuit has acquired a new function. It helps form the wing's eyespots, an antipredatory adaptation that arose after the insect body plan [65]. This example illustrates that a regulatory circuit may acquire additional functions incrementally via gradual genetic change. The order in which the mutations leading to a new function arise and go to fixation can have a profound impact upon the evolution of such phenotypes [66]. In particular, early mutations have the potential to influence the phenotypic effects of later mutations, which can lead to a phenomenon known as historical contingency.

We next ask whether it is possible for a circuit to incrementally evolve regulatory functions in any order, or whether this evolutionary process is susceptible to historical contingency. In other words, is it possible that some sequence of genetic changes that lead a circuit to have *k* functions also preclude it from gaining an additional function? The genotype space framework allows us to address this question in a systematic way, because it permits us to see contingency as a result of genotype set fragmentation. Specifically, contingency means that, as a result of fragmentation, the genotype network of a new function may become inaccessible from at least one of the genotype networks of a *k*-function's genotype set. To ask whether this occurs in our model regulatory circuits, we considered all 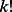 permutations of every *k*-function. These permutations reflect every possible order in which a circuit may acquire a specific combination of *k* functions through a sequence of genetic changes. To determine the frequency with which historical contingency occurs, we calculate the number of genotype networks per genotype set, as the *k* functions are incrementally added. This procedure is outlined in Fig. S4 and detailed in the Material and Methods section. We note that historical contingency is not possible when 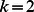 because all monofunctions comprise genotype sets with a single connected genotype network. Historical contingency is also not possible when 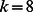, because there is only one genotype that yields this combination (Fig. 2).

**Figure 5:**
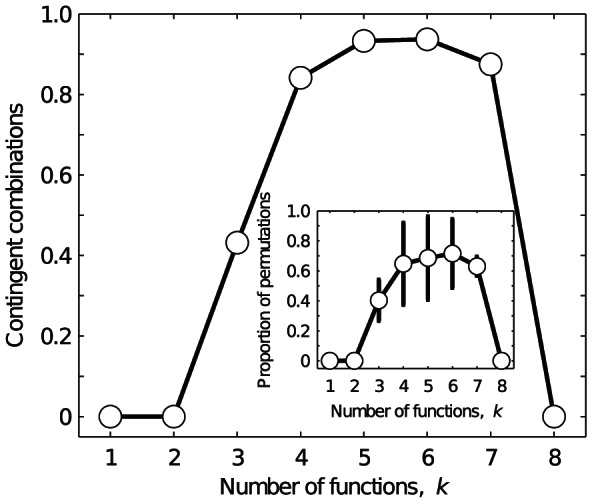
Historical contingency in multifunctional regulatory circuits. Each data point shows the proportion of combinations of *k* functions that exhibit contingency, as a function of *k*. The line is provided as a visual guide. The inset shows the average proportion of the 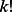 permutations of each combination of *k* functions that exhibit contingency. Error bars denote one standard deviation.

In Fig. 5, we show the relationship between the proportion of *k*-functions that exhibit historical contingency and the number of functions *k*. For as few as 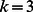 functions, 43% of all *k*-functions exhibit historical contingency. This percentage is highest for 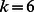, where 94% of combinations are contingent. The inset of Fig. 5 shows the proportion of the 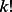 permutations of a *k*-function in which genotype set fragmentation may preclude the evolution of the *k*-function. Again, this proportion is highest for 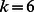 functions. These results highlight an additional constraint of multifunctionality. Not only does the number of genotypes with *k* functions decrease as *k* increases, but the dependence upon the temporal order in which these functions evolve tends to increase.

In the Supporting Online Material, we repeat the above calculations to show how our results scale to equilibrium expression states with period 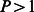 (For the sake of computational tractability, we restrict our attention to the case where all equilibrium expression states have the same period *P*). We show that the exponential decrease in the number of circuits with *k* functions also holds for periodic equilibrium expression states, but that the maximum number of functions per circuit decreases with increasing 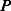 (Fig. S8). So long as 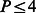, it is possible for a circuit to have more than one function. In this case, the inverse relationship between robustness to genetic perturbation and the number of functions *k* also holds (Fig. S9). Similarly, the results pertaining to genotype set fragmentation hold so long as 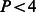 (Fig. S10). Lastly, the results pertaining to historical contingency only hold when 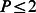. This is because it is not possible for a circuit with an equilibrium expression pattern of period 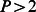 to have more than 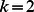 functions, which is a prerequisite for historical contingency (Material and Methods). Taken together, these additional observations show that the results obtained for fixed-point equilibrium expression states can also apply to periodic equilibrium expression states, so long as 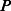 is not too large.

## Discussion

We have used a Boolean model of gene regulatory circuits to exhaustively characterize the functions of all possible combinations of circuit topologies and signal-integration functions in three-gene circuits. The most basic question we have addressed is whether multifunctionality is easy or difficult to attain in regulatory circuits. Our results show that while the number of circuits with *k* functions decreases sharply as *k* increases, there are generally thousands of circuits with *k* functions, so long as *k* is not exceedingly large. Thus, multifunctionality is relatively easy to attain, even in the tiny circuits examined here.

It is worth considering how this result might translate to larger circuits. In a related model of gene regulatory circuits with 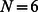 genes, the genotype sets of bifunctions comprised an average of 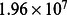 circuits [21], which is over an order of magnitude more circuits per bifunction than observed here (Fig. 3, inset). For a greater number of functions *k*, we expect the number of circuits per *k*-function to increase as the number of genes *N* in the regulatory circuit increases. This is because the maximum number of circuits with a given *k*-function is 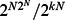, which is the total number of circuits with *N* genes (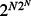) multiplied by the maximum proportion of circuits per multifunction (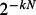). For a given number of functions *k*, this quotient will increase hyper-exponentially as *N* increases, indicating a dramatic increase in the maximum number of circuits per *k*-function. More generally, because the fractional size of a *k*-function's genotype set can be approximated as the product of the fractional sizes of the genotype sets of its *k* constituent monofunctions (Fig. S7) and because the total number of circuits increases exponentially with *N*, our observation that there are many circuits with *k* functions is expected to scale to larger circuits.

The next question we asked is whether there is a tradeoff between the robustness of a *k*-function and the number of functions *k*. We found that the robustness of a *k*-function decreases as *k* increases. However, some degree of robustness is generally maintained, so long as *k* is not too large. These observations suggest that the number of circuit functions generally does not impose severe constraints on the evolution of circuit genotypes, unless the number of functions is very large. Our current knowledge of biological circuits is too limited to allow us to count the number of functions per circuit. However, we can ask whether the functional “burden” on biological circuits is very high. If so, we would expect that the genes that form these circuits and their regulatory regions cannot tolerate genetic perturbations, and that they have thus accumulated few or no genetic changes in their evolutionary history. However, this is not the case. The biochemical activities and regulatory regions of circuit genes can diverge extensively without affecting circuit function [55], [59], [61], [67], and the very different circuit architectures of distantly related species can have identical function [24], [28]. Further, circuits are highly robust to the experimental perturbation of their architecture, such as the rewiring of regulatory interactions [20]. More indirect evidence comes from the study of genes with multiple functions, identified through gene ontology annotations. The rate of evolution of these genes is significantly but only weakly correlated with the number of known functions [68]. Thus, the functional burden on biological genes and circuits is not sufficiently high to preclude evolutionary change.

Previous studies of monofunctional regulatory circuits have revealed broad distributions of circuit robustness to genetic perturbation [16], [18], [29]. We therefore asked if this is also the case for multifunctional circuits. We found that circuit robustness was indeed variable, but that the mean and variance of the distributions of circuit robustness decreased as the number of functions *k* increased. Thus, variation in circuit robustness persists in multifunctional circuits, so long as *k* is not too large. This provides further evidence that robustness to mutational change may be considered the rule, rather than the exception, in biological networks [1], [18], [20], [29]. However, to make the claim that robustness to genetic perturbation is an evolvable property in multifunctional regulatory circuits requires not only variability in circuit robustness, but also the ability to change one circuit into another via a series of mutations that do not affect any of the circuit's functions.

We therefore asked whether it is possible to interconvert any two circuits with the same function via a series of function-preserving mutational changes. We showed that this is always possible for monofunctions, but not necessarily for multifunctions, because these often comprise fragmented genotype sets. Genotype set fragmentation has also been observed at lower levels of biological organization, such as the mapping from RNA sequence to secondary structure [69]. Such fragmentation has two evolutionary implications, as has recently been discussed for RNA phenotypes [70]. First, the mutational robustness of a phenotype (function) depends upon which genotype network its sequences inhabit, as we have also shown for regulatory circuits (Fig. S11). Second, it can lead to historical contingency, where the phenotypic effects of future mutations depend upon the current genetic background. Such contingency indeed occurs in our circuits, because the specific genotype network that a circuit (genotype) occupies may be influenced by the temporal order in which a circuit's functions (phenotypes) have evolved. This order in turn may affect a circuit's ability to evolve new functions.

These observations hinge on the assumption that the space between two (disconnected) parts of a fragmented genotype set is not easily traversed. For example, in RNA it is well known that pairs of so-called compensatory mutations can allow transitions between genotype networks [71], thus alleviating the historical contingency caused by fragmentation. To assess whether an analogous phenomenon might exist for regulatory circuits, we calculated the average distance between all pairs of genotypes on distinct genotype networks for circuits with the same *k*-function. We found that this distance decreases as the number of functions *k* increases, indicating an increased proximity between genotype networks (Fig. S12). However, those pairs of genotypes in any two different genotype networks that had the minimal distance of two mutations never exceeded 1% of all pairs of genotypes on these networks, and was as low as 0.03% for 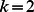 functions (Fig. S12A, inset). This means that transitions between genotype networks through few mutations are not usually possible in these model regulatory circuits. Thus, the multiple genotype networks of a genotype set can indeed be considered separate from one another.

Using a Boolean model of gene regulatory circuits comes with several caveats that are worth highlighting. First, the mutational distance between certain logical functions may not correspond to their distance in a biological context. For example, the signal-integration logic of a gene can mutate from an OR function to an XOR function by changing only a single bit. In contrast, research in synthetic biology suggests that these logical functions are separated by greater mutational distances. While the OR function can be encoded as a simple two-input circuit [37], the XOR function has necessitated cascading signals between distinct circuits [37] or cells [72], [73], or chemically-induced DNA inversions [74]. In some biological circuits, such as the *lac* operon in *E. coli*, it may not be possible to transform an OR function into an XOR function at all [32]. However, experimental investigations of the cis-regulatory codes of synthetic and natural circuits are far from exhaustive, and it is therefore possible that there exist alternative implementations of these logical functions that more closely resemble their Boolean representations [31]. Second, the model makes the simplifying assumptions that gene expression states are binary and that regulatory interactions are static. In biological circuits, gene expression is continuous and regulatory interactions are dynamic, varying in both time and space. Despite these limitations, the assumption of binary expression often provides a reasonable approximation [32] and numerous studies have demonstrated the model's ability to precisely replicate the expression dynamics of biological circuits, even under the assumption of static regulatory interactions [50]–[53]. Third, we assume that gene states are updated synchronously [49], which is clearly not the case in biological circuitry. Asynchronous updating can affect the transient dynamics of a circuit [75] and its equilibrium expression patterns [76], and may therefore impact circuit function. This becomes especially problematic when the equilibrium expression pattern is periodic [77]. However, the fixed-point equilibrium expression states of Boolean circuits do not vary between asynchronous and synchronous updating schemes [78], so we did not consider asynchronous updating. While it is possible that some of our results depend upon this assumption, we stress that this study could not have been performed without it. The exhaustive enumeration of genotype space is not computationally feasible under asynchronous updating because all possible orderings of updates have to be considered for each genotype. Fourth, we did not explicitly consider gene expression noise. While this is an important aspect of genetic regulation [79], robustness to gene expression noise is correlated with robustness to genetic perturbation in model regulatory circuits [18]. Thus, we used the latter as a proxy for the former. Lastly, we only considered small, three-gene circuits. This allows for the exhaustive enumeration of all possible circuit topologies and signal-integration functions, but limits the direct applicability of our results to similarly sized circuits. However, we expect our results to also apply to larger circuits, as we have discussed. We emphasize that our observations are not derived from one circuit and its functions, but from an enormous circuit space, comprising a class of circuits that capture biological phenomena in diverse organisms.

## Materials and Methods

### Model details

We consider fully connected Boolean circuits with 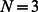 genes. The binary state 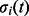 of a gene *i* at time *t* is a function 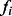 of the states of all 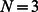 genes at time 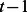:
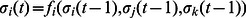
(1)The function 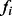 maps all of the 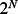 possible combinations of input expression states to an output expression state. This function represents the gene's signal-integration logic and can be represented as a look-up table (Fig. 1A). The circuit is initialized with an initial expression state 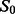 and all genes are updated synchronously according to their individual functions *f* until a steady-state expression pattern 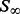 is reached. The expression pattern 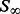 can be a fixed-point (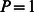) or a cycle (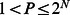).

The update functions *f* of all *N* genes can be represented as a single vector of length 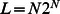 (Fig. 1B). We measure the equilibrium expression states 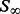 for all 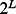 possible vectors for each of the 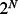 possible initial expression states 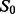. In doing so, we not only enumerate all signal-integration functions, but also all circuit topologies. This is because some functions *f* make a gene independent of one or more of its *N* regulatory inputs. For example, in Fig. 1A, the regulatory interaction 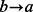 is inactive because for any combination of regulatory inputs, the expression state of gene *a* is unaffected by the expression state of gene *b*.

### Dynamic regimes of Boolean circuits

Boolean circuits exhibit three dynamic regimes that have been called ordered, critical, and chaotic [49]. The ordered regime is characterized by a general insensitivity to perturbation that results from having few equilibrium states, each with large basins of attraction, whereas the chaotic regime is characterized by extreme sensitivity to perturbation that results from having many equilibrium states with small basins of attraction. The critical regime lies at the interface of these two extremes. Several studies have focused on characterizing the dynamic regimes of biological circuits [80]–[83] and on understanding how these regimes influence circuit dynamics *in silico* [49], [84].

The dynamic regime of a circuit can be determined by calculating its sensitivity 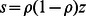, where *z* is the average number of regulators per gene and 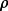 is the average probability of gene expression per gene (*i.e.*, the proportion of the genotype *G* that is nonzero) [85], [86]. The ordered regime corresponds to 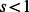, the critical regime to 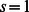, and the chaotic regime to 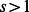. Since 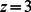 for all circuits considered here, the dynamic regime is determined solely by 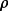.

### Multifunctions and their combinations

The maximum number of functions a circuit can produce is 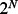 because we require the equilibrium expression states of any multifunction 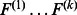 to be unique (*i.e.*, 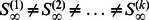). We also require that the initial expression states are unique (*i.e.*, 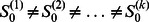). While the deterministic nature of the model makes this latter requirement superfluous — different equilibrium states require different initial states — we specify it to highlight the fact that each function pertains to a specific input signal, which may differ between environments or tissue-specific conditions.

A circuit may produce various combinations of *k* functions, as shown in Fig. 1. We note that some combinations of functions are not feasible. As an example, consider a hypothetical combination 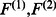 where 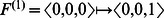, 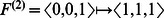. This combination is not feasible because the equilibrium expression state of 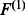 is a transient state of 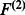.

Our usage of the word *function* differs from existing terminology for describing the mapping of initial to equilibrium states in Boolean circuits. For a given circuit, an *attractor* is an equilibrium state (fixed-point or periodic) that can be reached from at least one initial state. An attractor's *basin of attraction* is the set of initial states that lead to that attractor. The *attractor landscape* is the set of all attractors and their basins of attraction. These terms are distinct from our use of the words *function* and *k-function*, which are concerned with specific pairs of initial and equilibrium states, because specific initial states provide key inputs to most biological circuits in development and physiology. The only equivalence between terms occurs when 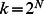. Such a *k*-function is equivalent to the circuit's attractor landscape, because each of the 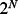 initial states map onto themselves. In this case, the entire attractor landscape is embodied in the function.

### Robustness

We measure the robustness of circuits and of *k*-functions. The robustness of a circuit is calculated as the proportion of its mutational neighbors that have the same *k*-function, as follows. First, we remove the entries in the circuit's genotype *G* that correspond to inactive regulatory interactions. This results in a new vector 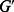 that may differ from *G*. Second, we determine the fraction of single mutants of 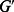 that produce the same multifunction. This is achieved by flipping each bit in 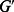, one at a time, and determining whether the resulting genotype has the same *k*-function. We refer to this measure of circuit robustness as 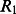, which is the measure that is used throughout the main body of the text. The robustness of a *k*-function is calculated as the average robustness of all circuits with that *k*-function.

Alternatively, the robustness of a circuit can be calculated as the connectivity of its genotype *G* in a genotype network of a *k*-function, divided by the maximum possible connectivity *L*. We refer to this measure of circuit robustness as 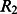. In Fig. S13, we show that these two calculations result in measures of *k*-function robustness that are highly correlated (Spearmans 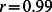). The fact that the data are always below the identity line indicates that 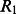 is a more conservative measure of robustness than 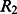.

### Historical contingency

To detect whether a combination of *k* functions may exhibit historical contingency, we consider all 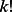 permutations of those functions. We define a combination of *k* functions to be contingent if there exists at least one permutation that violates, and at least one other permutation that satisfies, the following condition: For the functions 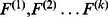 in the permutation, there exists a 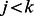 such that the number of genotype networks in the genotype set of function 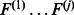 is greater than the number of genotype networks in the genotype set of function 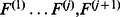. For example, in Fig. S4, the permutation 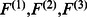 satisfies this condition because the genotype set of 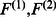 comprises two genotype networks while the genotype set of 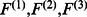 comprises only one genotype network. All other permutations violate this condition. Therefore this combination of *k*-functions exhibits historical contingency. Since all monofunctions comprise a single, connected genotype network, it is impossible for any bifunction to satisfy the condition above. Thus, in these model regulatory circuits, historical contingency can only occur for 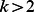.

## Supporting Information

Figure S1 **Robustness and genotype set size.** Each data point shows the size of a specific *k*-function's genotype set as a function of its robustness. Symbol types correspond to the number of functions *k* in the *k*-function. Note the logarithmic scale of the y-axes.
(EPS)

Figure S2
**Genotypic robustness is an evolvable property of multifunctional circuits.** Each data point shows genotypic robustness before and after 1000 steps of a random walk. In each step of the random walk, robustness is not allowed to decrease. Each panel shows data for 1000 separate random walks on genotype networks of multifunctions for (A) 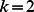, (B) 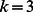, and (C) 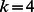 functions. These are the same genotype networks used in Fig. 3B–D, respectively. Since all points lie on or above the identity line, it is always possible to increase robustness via a series of mutations that preserve the *k*-function, unless the initial genotype already resides atop a local robustness peak. The y-axis label of (A) applies to all panels.(EPS)

Figure S3
**Most circuits are chaotic, regardless of dynamic regime.** Each panel shows the number of circuits with sensitivity *s* for (A) 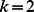, (B) 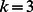, and (C) 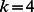 functions. Circuit sensitivity is used to determine a circuit's dynamic regime, as indicated by the white and shaded regions. The line separating these regions corresponds to the so-called critical regime. For each *k*, we show data for the same genotype networks shown in Fig. 3B–D.(EPS)

Figure S4
**Schematic illustration of genotype set fragmentation and historical contingency.** In each panel, the open circles represent circuits and the shading represents circuit function. Three functions are shown, as indicated by the legend. Two circuits are neighbors (connected by a solid line) if they have the same *k*-function and their genotypes *G* differ by a single regulatory element (Fig. 1D). Each panel corresponds to a different *k*-function, and *k* increases as the three columns of the figure are read from left to right (A: 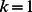, B,C: 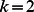, D: 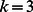). (A) The genotype set of the monofunction 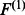 comprises a single connected genotype network, meaning that any genotype can be reached from any other via a series of small genetic changes that do not alter circuit function. (B) The bifunction 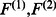 shows an example of fragmentation, where the genotype set comprises two isolated genotype networks, despite the fact that the genotype sets of the two constituent monofunctions comprise single, connected genotype networks. This means that some genotypes with this bifunction cannot be reached from some others via a series of function-preserving genetic changes. (C) In contrast, the genotype set of the bifunction 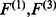 comprises a single, connected genotype network. (D) This example shows one possible multifunction with all 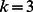 functions. Its genotype set is also made up of a single, connected genotype network. However, there are six possible orderings in which this multifunction could evolve and two of these are shown in panels A, B, and D (upper sequence of arrows 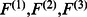), as well as in panels A, C, and D (lower sequence of arrows, 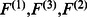). This provides an illustration of historical contingency because the order in which the functions evolve dictates whether or not it is possible to evolve all functions. Specifically, the genotype set fragmentation shown in (B) may confine a population to the upper right genotype network, precluding navigation to the region in genotype space where all 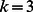 functions can be satisfied.(EPS)

Figure S5 **An example of genotype set fragmentation.** The genotype set of the bifunction shown in (A) is fragmented into five genotype networks. Specifically, this genotype set consists of one large genotype network with 11,380 genotypes and four small genotype networks that each comprise a single genotype. In (B–F), we show genotypes from these five genotype networks and their corresponding “trajectories” from initial to equilibrium states. The genotype in (B) is part of the large genotype network and was chosen because it has the same “trajectory length” as those genotypes in (C–F), which come from the four small genotype networks. Note that any mutations to the genotypes in (C–F) will destroy the bifunction, and it is therefore not possible to reach the large genotype network via a series of function-preserving mutations. We note that at present, we do not have an analytical explanation for the phenomenon of genotype set fragmentation. This presents an exciting direction for future research.
(EPS)

Figure S6
**Cumulative distributions of the fractional sizes of the genotype networks of** ***k*** **-functions.** Distributions are shown for 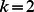, 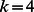, and 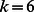 functions. The vertical dashed lines indicate the maximum fractional size of a genotype network for monofunctions 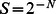 [44], raised to the *k*th power. Note the logarithmic scale of the x-axis.(EPS)

Figure S7
**Approximating the fractional size of a genotype set.** The product of the fractional sizes 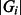 of the genotype sets 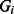 of monofunctions can serve as an order-of-magnitude approximation for the fractional sizes of the genotype sets of multifunctions. Here we show all 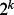 possible arrangements of genotype set sizes for (A) 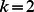 and (B) 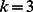 functions.(EPS)

Figure S8
**Multifunctional regulatory circuits with multi-state equilibria** 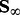 **of period** ***P*** **.** Each data point shows the proportion and number of genotypes with *k* functions. The lines are provided as a visual guide. The period *P* of 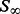 increases as the lines are read from right to left. The maximum number of functions per circuit is dictated by *P*. For example, if 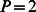 then a circuit can have at most 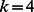 functions as this accounts for all possible 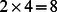 expression states. Note the logarithmic scale of the y-axes.(EPS)

Figure S9
**Robustness of** ***k*** **-functions with multi-state equilibria** 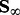 **of period** ***P*** **.** Each data point corresponds to the genotype set of a specific *k*-function with equilibrium states of period (A), 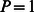, (B), 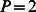, (C), 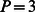, (D), 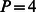, (E), 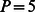, (F), 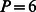, (G), 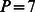, (H), 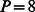. For 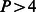, it is not possible for a circuit to have more than one function because we require that the states in 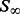 are all unique (Material and Methods). The axes labels of the inset are the same as in Fig. 3. Note the logarithmic scale of the y-axis.(EPS)

Figure S10
**Genotype set fragmentation for** ***k*** **-functions with multi-state equilibria** 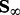 **of period** ***P*** **.** Each data point shows the number of genotype networks in the genotype set of a *k*-function with equilibrium states of period (A), 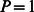, (B), 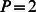, (C), 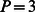, (D), 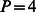, (E), 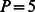, (F), 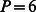, (G), 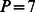, (H), 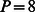. The insets show the proportion of genotype sets that comprise a single genotype network, as a function of *k* (cf. Fig. 4).(EPS)

Figure S11
**The average robustness of a circuit may vary between the genotype networks of a** ***k*** **-function's genotype set.** Each panel corresponds to a *k*-function with the largest number of genotype networks in its genotype set for (A) 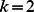, (B) 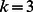, and (C) 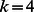. Each panel depicts a histogram of the average circuit robustness per genotype network. Those genotype networks with an average circuit robustness of zero comprise a single genotype.(EPS)

Figure S12
**Transitions between genotype networks are rare in model gene regulatory circuits.** For the genotype set of each *k*-function, we calculated the mutational distance between all pairs of genotypes that inhabited distinct genotype networks. Each data point in (A) depicts the average of this measure across all *k*-functions. Error bars denote one standard deviation, but are typically smaller than the symbol size. Data only exists for 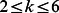, because these are the only values of *k* for which genotype set fragmentation occurs (Fig. 4). The inset of (A) shows the proportion of all pairs of genotypes from distinct genotype networks of the same *k*-function that were separated by a mutational distance of two. Representative distributions of mutational distance for the genotype sets of multifunctions with (B) 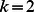, (C) 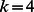, and (D) 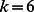 are also provided.(EPS)

Figure S13
**Alternative calculations of circuit robustness yield similar measures of** ***k*** **-function robustness.** Each data point depicts the robustness of a *k*-function as measured using 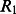 and 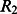 (Material and Methods), revealing a strong correlation between these measures (Spearman's 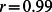). The identity line is shown for reference.(EPS)

Table S1 **The possible compositions of** ***k*** **-functions.** The numbers in the brackets denote *I* and *T*, respectively. Note that a circuit encounters one state for each *I* and at least two states for each *T*.
(PDF)

Text S1 **Analytical results.** This section provides analytical solutions for the number of circuits per *k*-function, the number of unique *k*-functions, and the number of *k*-functions with zero robustness. Additionally, we show analytically why there are no “prohibited” *k*-functions.
(PDF)
