# Supplementary material for: Constraint and Contingency in Multifunctional Gene Regulatory Circuits
Source: PLoS Comput Biol. 2013 Jun 6;9(6):e1003071. doi: 10.1371/journal.pcbi.1003071 (PMC3675121; doi:10.1371/journal.pcbi.1003071)
Supplement: Table S1 — The possible compositions of k -functions. The numbers in the brackets denote I and T, respectively. Note that a circuit encounters one state for each I and at least two states for each T. (PDF) [file pcbi.1003071.s014.pdf]

k	Number of compositions	Compositions
1	2	$\langle 1, 0\rangle$, $\langle 0, 1\rangle$
2	3	$\langle 2, 0\rangle$, $\langle 1, 1\rangle$, $\langle 0, 2\rangle$
3	4	$\langle 3, 0\rangle$, $\langle 2, 1\rangle$, $\langle 1, 2\rangle$, $\langle 0, 3\rangle$
4	5	$\langle 4, 0\rangle$, $\langle 3, 1\rangle$, $\langle 2, 2\rangle$, $\langle 1, 3\rangle$, $\langle 0, 4\rangle$
5	4	$\langle 5, 0\rangle$, $\langle 4, 1\rangle$, $\langle 3, 2\rangle$, $\langle 2, 3\rangle$
6	3	$\langle 6, 0\rangle$, $\langle 5, 1\rangle$, $\langle 4, 2\rangle$
7	2	$\langle 7, 0\rangle$, $\langle 6, 1\rangle$
8	1	$\langle 8, 0\rangle$
