# Supplementary material for: Constraint and Contingency in Multifunctional Gene Regulatory Circuits
Source: PLoS Comput Biol. 2013 Jun 6;9(6):e1003071. doi: 10.1371/journal.pcbi.1003071 (PMC3675121; doi:10.1371/journal.pcbi.1003071)
Supplement: Text S1 — Analytical results. This section provides analytical solutions for the number of circuits per k-function, the number of unique k-functions, and the number of k-functions with zero robustness. Additionally, we show analytically why there are no “prohibited” k-functions. (PDF) [file pcbi.1003071.s015.pdf]

Supplementary results

Analytical determination of the number of circuits per k-function

Here we analytically determine the number of circuits per k-function for three-gene circuits. This number, which we also refer to as genotype set size, depends on the number of unique states each circuit encounters in mapping its initial states to equilibrium states. As in the main text, we only consider functions where S_∞ is a fixed-point. We refer to functions with $S_0 = S_\infty$ as identity functions, and to functions with $S_0 \neq S_\infty$ as transition functions.

For a given k-function, let I denote the number of identity functions, and let T denote the number of transition functions. We use the notation $\langle I, T\rangle$ to represent the composition of a k-function. For example, the bifunction $F^{(1)} : (\langle 0, 0, 0\rangle \mapsto \langle 0, 0, 0\rangle)$, $F^{(2)} : (\langle 0, 0, 1\rangle \mapsto \langle 0, 1, 1\rangle)$ has $I = 1$ and $T = 1$. Its composition is therefore represented as $\langle 1, 1\rangle$. Similarly, the composition of a trifunction with $I = 2$ and $T = 1$ is represented as $\langle 2, 1\rangle$. Each k-function has a only limited number of possible compositions, due to the finite number of possible states 2^N (Table S1). For each value of k, this number of possible compositions is identical to the number of unique genotype set sizes shown in the inset of Fig. 3. This already hints that the number of circuits per k-function depends solely upon the k-function's composition. Below we show that this is indeed the case, by explaining how the number of circuits with a given k-function can be enumerated exactly.

We explain this enumeration for the simplest case, by considering the two genotype set sizes of monofunctions (Fig. 3, inset). If the composition of the monofunction is $\langle 1, 0\rangle$, then each circuit in the genotype set assumes only a single state in its "trajectory" from the initial state to the final state, namely the initial state itself ($S_0 = S_\infty$). This trajectory is dictated by only N entries of the genotype G, because each unique circuit state corresponds to exactly one entry in each gene's signal-integration logic (Fig. 1A,B). This means that the other $L - N$ entries of G can take on any value. The corresponding genotype set size is therefore 2^{L-N}. Its fractional size is $2^{L-N}/2^L = 2^{-N}$.

If the composition of the monofunction is $\langle 0, 1\rangle$, then each circuit in the genotype set assumes more than one state in its trajectory from the initial state to the final state. In principle, each circuit may encounter between 2 and 2^N states. If a circuit only encounters two states (*i.e.*, $S_0 \to S_\infty$), then $2N$ entries of the genotype dictate this trajectory and the other $L - 2N$ entries can take on any value. Therefore there are 2^{L-2N} circuits that use 2 states to go from S_0 to S_∞. Similarly, if a circuit assumes

three states (*i.e.*, $S_0 \to S_1 \to S_\infty$), then $3N$ entries of the genotype dictate this trajectory and the other $L - 3N$ entries can take on any value. Since there are $2^N - 2 = 6$ choices for S_1, there are $6 \times 2^{L-3N}$ circuits that use 3 states to map S_0 to S_∞. Similarly, there are $6 \times 5 \times 2^{L-4N}$ circuits that use four states, because there are 6 choices for S_1 and 5 choices for S_2. By extending this argument, it is clear that if a trajectory from S_0 to S_∞ involves $a > 1$ states, then there are

$$\frac{(2^N - 2)!}{(2^N - a)!} 2^{L-aN} \tag{1}$$

monofunctional circuits with that trajectory. Thus, there are

$$\sum_{a=2}^{2^N} \frac{(2^N - 2)!}{(2^N - a)!} 2^{L-aN} \tag{2}$$

monofunctional circuits with the composition $\langle 0, 1 \rangle$.

The same kind of argument allows us to derive the number of circuits S for any k-function of composition $\langle I, T \rangle$. For circuits with $N = 3$ genes, this number is given by

$$S(I,T) = \begin{cases} 2^{L-IN}, & \text{if } T = 0 \text{ and } 0 < I \leq 8 \\ \sum\limits_{a=2}^{2^N-I} \frac{(2^N-I-2T)!}{(2^N-I-a)!} 2^{L-IN-aN}, & \text{if } T = 1 \text{ and } 0 \leq I \leq 6 \\ \sum\limits_{a=2}^{2^N-I} \sum\limits_{b=2}^{2^N-I-a} \frac{(2^N-I-2T)!}{(2^N-I-a-b)!} 2^{L-IN-(a+b)N} & \text{if } T = 2 \text{ and } 0 \leq I \leq 4 \\ \sum\limits_{a=2}^{2^N-I} \sum\limits_{b=2}^{2^N-I-a} \sum\limits_{c=2}^{2^N-I-a-b} \frac{(2^N-I-2T)!}{(2^N-I-a-b-c)!} 2^{L-IN-(a+b+c)N} & \text{if } T = 3 \text{ and } 0 \leq I \leq 2 \\ \sum\limits_{a=2}^{2^N-I} \sum\limits_{b=2}^{2^N-I-a} \sum\limits_{c=2}^{2^N-I-a-b} \sum\limits_{d=2}^{2^N-I-a-b-c} \frac{(2^N-I-2T)!}{(2^N-I-a-b-c-d)!} 2^{L-IN-(a+b+c+d)N} & \text{if } T = 4 \text{ and } I = 0. \end{cases} \tag{3}$$

We compared the number of circuits with k-functions of composition $\langle I, T \rangle$ enumerated through this combinatorial argument to the same number obtained through our exhaustive numerical enumeration of circuit space. The two numbers coincide exactly for all values of $\langle I, T \rangle$ in Table S1, demonstrating that our analytical calculations are correct.

Analytical determination of the number of unique *k*-functions

Next we analytically determine the number of unique k-functions, motivating our approach with two simple examples and using the terminology developed in the preceding section.

In the first example, we consider monofunctions ($k = 1$). These can have one of two compositions: $\langle 1, 0\rangle$ or $\langle 0, 1\rangle$ (Table S1). In the former case, there are $\binom{2^N}{1} = 8$ unique possibilities, each corresponding to a different identity function. In the latter case, there are 8 possible choices for the initial state and 7 possible choices for the equilibrium state resulting in $P_2^{2^N} = 56$ unique possibilities, where P denotes the permutation function. Thus, there are $8 + 56 = 64$ possible monofunctions.

In the second example, we consider bifunctions ($k = 2$). These can have one of three compositions: $\langle 2, 0\rangle$, $\langle 1, 1\rangle$, or $\langle 0, 2\rangle$ (Table S1). For bifunctions of composition $\langle 2, 0\rangle$, there are $\binom{8}{2} = 28$ unique combinations of two identity functions. For bifunctions of composition $\langle 1, 1\rangle$, there are $\binom{8}{1} = 8$ choices for the identity function, leaving $P_2^7 = 42$ choices for the transition function. This results in $8 \times 42 = 336$ unique possibilities. Finally, for bifunctions of composition $\langle 0, 2\rangle$, there are $P_2^8 = 56$ choices for the first transition function and $P_2^6 = 30$ choices for the second transition function. This results in $56 \times 30/2 = 840$ unique possibilities, where the denominator accounts for the fact that the order of the two functions does not matter. Thus, there are $28 + 336 + 840 = 1204$ possible bifunctions.

This same reasoning can be used to determine the number K of k-functions with any composition $\langle I, T\rangle$. This number is given by

$$K(I, T) = \frac{\binom{2^N}{I} \prod_{t=0}^{T-1} P_2^{2^N - I - 2t}}{T!}. \tag{4}$$

This equation can be used to determine the number of k-functions for any k by summing over all possible compositions of k functions (Table S1). We have performed this calculation and the numbers match the results of our exhaustive enumeration (Fig. 2, inset) exactly.

The size of genotype space is equivalent to the number of unique attractor landscapes

The number of unique attractor landscapes A can be calculated as the number of possible directed graphs with $V = 2^N$ vertices, in which each vertex has only a single outgoing edge. Each vertex in each graph represents a circuit state, and directed edges denote transitions between circuit states. This number is

$A = V^V = 2^{N2^N}$, which is equivalent to the number of genotypes. Since each genotype corresponds uniquely to one of these directed graphs, and thus uniquely to one attractor landscape, it immediately follows that there must be at least one genotype for each k-function. This explains why the analytically determined number of k-functions $K(I,T)$ (solid line in Fig. 2, inset) is the same as the number of k-functions observed through exhaustive enumeration (open circles in Fig. 2, inset).

Analytical determination of the number of *k*-functions with zero robustness

Some k-functions comprise only one genotype, which means they have zero robustness. This occurs when all 2^N circuit states are included as initial or equilibrium states in the k-function. This implies that k-functions with zero robustness can only occur if $k \geq 2^N/2$, because this is the smallest value of k for which all 2^N states can be involved in the k-function. This explains why in Fig. 3 we only see k-functions with zero robustness for $k \geq 4$. For example, when $k = 4$, only multifunctions with composition $\langle 0, 4 \rangle$ have zero robustness.

The number of k-functions with zero robustness R_0 can be calculated as

$$R_0(k) = \binom{2^N}{k} \frac{k!}{(2k - 2^N)!}, \tag{5}$$

where the first term captures the number of ways to choose the equilibrium states and the second term captures the number of ways to choose the remaining initial states. We have confirmed that $R_0(k)$ matches the number of k-functions with zero robustness as determined through exhaustive enumeration (Fig. 3).
